# Fungal Endophyte (*Epichloë festucae*) Alters the Nutrient Content of *Festuca rubra* Regardless of Water Availability

**DOI:** 10.1371/journal.pone.0084539

**Published:** 2013-12-18

**Authors:** Beatriz R. Vázquez-de-Aldana, Antonia García-Ciudad, Balbino García-Criado, Santiago Vicente-Tavera, Iñigo Zabalgogeazcoa

**Affiliations:** 1 Department of Abiotic Stress, Instituto de Recursos Naturales y Agrobiología de Salamanca (IRNASA), Consejo Superior de Investigaciones Científicas (CSIC), Salamanca, Spain; 2 Department of Statistics, Universidad de Salamanca, Salamanca, Spain; Institute for Plant Protection (IPP), CNR, Italy

## Abstract

*Festuca rubra* plants maintain associations with the vertically transmitted fungal endophyte *Epichloë festucae*. A high prevalence of infected host plants in semiarid grasslands suggests that this association could be mutualistic. We investigated if the *Epichloë*-endophyte affects the growth and nutrient content of *F. rubra* plants subjected to drought. Endophyte-infected (E+) and non-infected (E−) plants of two half-sib lines (PEN and RAB) were subjected to three water availability treatments. Shoot and root biomass, nutrient content, proline, phenolic compounds and fungal alkaloids were measured after the treatments. The effect of the endophyte on shoot and root biomass and dead leaves depended on the plant line. In the PEN line, E+ plants had a greater S:R ratio than E-, but the opposite occurred in RAB. In both plant lines and all water treatments, endophyte-infected plants had greater concentrations of N, P and Zn in shoots and Ca, Mg and Zn in roots than E- plants. On average, E+ plants contained in their shoots more P (62%), Zn (58%) and N (19%) than E- plants. While the proline in shoots increased in response to water stress, the endophyte did not affect this response. A multivariate analysis showed that endophyte status and plant line impose stronger differences in the performance of the plants than the water stress treatments. Furthermore, differences between PEN and RAB lines seemed to be greater in E- than in E+ plants, suggesting that E+ plants of both lines are more similar than those of their non-infected version. This is probably due to the endophyte producing a similar effect in both plant lines, such as the increase in N, P and Zn in shoots. The remarkable effect of the endophyte in the nutrient balance of the plants could help to explain the high prevalence of infected plants in natural grasslands.

## Introduction

Successful plant adaptation to a changing environment might occur as a result of existing intrinsic plant traits. In addition, the symbiotic microbiome of plants is receiving a growing attention because of its role as a source of complementary characteristics available for plant adaptation [1]. Endophytic fungi are an important component of the plant microbiome. These fungi are capable of infecting their hosts without causing apparent symptoms and are ubiquitous throughout the plant kingdom [2]. Many, possibly thousands of fungal species can behave as endophytes, displaying a wide variety of life cycles and types of associations with plants [3-5]. Particularly interesting are the mutualistic associations where plant adaptation to stressful conditions is improved by endophytes [6-9]. 

One of the most studied systems of plant-endophyte associations is that of the *Epichloë* (*Ascomycota*, Fam. *Clavicipitaceae*) and related asexual *Neotyphodium* species. These epichloid endophytes infect the aerial tissues, but not the roots, of several economically important turf and forage cool season grasses such as *Lolium perenne* [10]. During the vegetative phase of the host grass, systemic hyphae colonize the intercellular space of leaves, and infected plants do not show any obvious symptoms. When infected plants enter their reproductive phase, endophytic hyphae reach the flowering stems and colonize the seeds. With this type of vertical transmission the fungus is efficiently transmitted to plant offspring. Vertically transmitted endophytes like all *Neotyphodium* and some *Epichloë* species (i.e. *Epichloë festucae*) are symptomless during the reproductive phase of host plants. However, some species like *Epichloë typhina* are pathogenic during the host plant reproductive phase, when a fungal stroma develops around the developing plant inflorescences, blocking their emergence, and thus sterilizing the plant host. This condition is known as choke disease [11]. Pathogenic species are horizontally transmitted to new host plants by means of sexual ascospores that develop on the perithecial stromata, and after their ejection can infect developing seeds from healthy plants. In contrast, the reproduction of asymptomatic fungal species is asexual and relies on vertical transmission to the seed progeny of infected plants. 

For long, asymptomatic endophyte-grass interactions have been considered as a defensive mutualism [10,12]. Asymptomatic epichloid endophytes of grasses are well known for the antiherbivore activity they supply to the host plant, thereby defending their own resources. Resistance to herbivores including mammals, insects and nematodes is mediated by a range of alkaloids produced by the endophyte within the host plant [13-15]. For example, ergovaline is toxic to grazing mammals and peramine is a feeding deterrent to insects [14]. On the other hand, the host provides food, shelter and means for reproduction and dispersal of the fungus. Furthermore, endophytes may improve host tolerance to abiotic stresses such as drought and metal toxicity [16]. 

Nevertheless, subsequent studies have shown that the endophyte effect on host plants is dependent on the plant and fungal genotypes and the environmental conditions. For example, the host plant and fungal genotype determine the endophyte effect on plant growth [17-21], plant nutrient content [22,23] or seed survival [24,25]. Thus, at the present time plant-endophyte interactions are considered to be variable and to range from antagonistic to mutualistic [3,12,26,27].

The effects of epichloid endophytes on plant performance under water-limiting conditions are still a subject of debate [28,29]. Early studies were made with a single cultivar of tall fescue (*Lolium arundinaceum= Festuca arundinacea*), and reported a positive effect of *Neotyphodium coenophialum* in plant growth under drought [30-32], but subsequent studies detected variable responses in different plant and fungal genotypes [17,33]. In perennial ryegrass (*Lolium perenne*) subjected to drought, the effects of *Neotyphodium lolii* are also variable [18,20,34-36]. In other hosts of epichloid endophytes, the effect of water stress has been less studied. For instance, no benefit of endophyte infection, in terms of biomass production, was found in plants of *Festuca rubra* or *Festuca pratensis* under drought stress [37]. To identify strong endophyte effects on plants, particularly if they seem to be independent on genotypes, is an important step towards understanding the often observed high prevalence of infected plants in natural environments.

Most studies on the effects of endophytic symbioses on drought have focused on plant biomass production and number of tillers. Although mechanisms of endophyte-mediated drought tolerance are not understood, there is some evidence that endophytes can modify some drought related physiological parameters in host plants [16]. One of the most common drought tolerance strategies in plants is the overproduction of solutes (i.e. proline, sugars, organic acids, calcium, potassium or chloride ions) that provide osmotic adjustment, an active accumulation of solutes that allow plants to maintain cell turgidity under water deficits [38,39]. The effect of epichloid endophytes on proline content of plants under drought is variable according to plant genotype and level of water stress [17,23,36]. Other plant secondary metabolites like phenols are associated with functions related to acclimation to stressful environments [40], and endophytic fungi have been shown to increase the production of phenolic compounds in host plants [41-43]. In addition, some evidence indicates that fungal alkaloids could protect grass-hosts from water stress [36,44].

*Festuca rubra* (red fescue) is a perennial grass tolerant to a wide range of ecological conditions and one of the most important turfgrass species in temperate regions [45]. In natural populations of *F. rubra* from diverse ecosystems across Europe, plants are often colonized by the endophyte *Epichloë festucae* [46-51]. Most *F. rubra* host plants are asymptomatic and produce infected seeds, although a few plants might develop choking stromata in some flowering stems [46,48]. *Festuca rubra* is a common species in savannah-like semiarid grasslands of western Spain (dehesas), a center of diversity of fine fescues [52]. In these grasslands about 70% of the *F. rubra* plants are colonised by *Epichloë festucae*, and the occurrence of choking stromata is very rare [48,53]. Here, we investigated whether the responses of the native *F. rubra* to endophyte infection depend on water availability. Based on these high infection rates observed in semiarid dehesa grasslands, our hypothesis was that *F. rubra* plants infected by *Epichloë* could be more tolerant of water stress than non-infected plants. Specifically, we addressed the following questions: (1) Does the endophyte improve the drought tolerance of the grass host? (2) Does the endophyte modify plant growth, nutrient content, proline and/or phenolic compounds of the grass host? (3) Does plant genotype affect the endophyte effect on the host grass?

## Materials and Methods

### Plant material

*Festuca rubra* (red fescue) is a perennial grass frequent in the dehesa grasslands of western Spain. Dehesas are savanna-like ecosystems featuring low-density *Quercus ilex* L. subsp. *ballota* trees in natural grasslands of a complex floristic composition [50]. This land is mainly used for the free-range grazing of beef cattle and fighting bulls but also Iberian pigs, sheep and wild animals occur. The climate is supra-Mediterranean, with cold winters and dry, warm summers. The average annual rainfall is 580 mm, seasonally distributed with 145 mm in spring, 37 mm in summer, 243 mm in the fall, and 155 mm in winter [54].

Two half-sib lines of *F. rubra* (PEN and RAB), each consisting of endophyte-infected (E+) and endophyte free (E−) plants were used for the experiment. Each line was developed from a single plant originally infected by *E. festucae*. The mother plants were collected at La Peña (PEN) and Raboso (RAB), two dehesa grasslands located 74 km apart in the province of Salamanca (western Spain). La Peña (41° 10´ 13´´N; -6° 31´19´´ W; 616 m a.s.l.) has a granitic substrate and Raboso (40° 32´ 38´´N; 6° 36´47´´ W; 590 m a.s.l.) has a sedimentary substrate. The owners of both grasslands gave us permission to collect plants. Both locations have a mean annual precipitation of about 600 mm (data based on unpublished data of a 10-year period). Asymptomatic plants were collected at the reproductive stage, and their infection status was verified by microscopic analysis of stem pith scrapings. The diagnosis by microscopy was verified by isolation of the fungus *Epichloë festucae* from plant stems and leaf sheaths in Petri dishes containing potato dextrose agar (PDA) [55]. In wild populations of *F. rubra* in grasslands similar to La Peña and Raboso, the prevalence of plants infected by *E. festucae* range from 44% to 92%, averaging 70% [48]. 

One infected plant from each location was transplanted to a pot containing a mixture of peat moss and perlite, and allowed to undergo vegetative growth for one month. Then, each plant was divided into several ramets, which were transplanted into 75-ml pots and maintained in a growth chamber with a 16 h-light photoperiod at 25°C. The endophyte was eliminated from half of the ramets of each plant by treatment with three doses of 400 μg of propiconazole (TILT, 400 mg a.i. l^−1^, CIBA), a systemic fungicide. The first and third doses were applied to the soil, and the second one was a foliar application. Fungicide treatments were spaced by 10 days between applications. The E- status of fungicide treated ramets was verified by plating surface sterilized leaf sheaths on PDA [55]. Treated and untreated plants were then transplanted to the field in a research farm near Salamanca. From these plants, E+ (infected) and E- (uninfected) seeds were obtained. Thus, E- seeds were the progeny obtained from clones of infected plants treated with a fungicide. *Festuca rubra* is an allogamous species, therefore, seeds produced from E+ or E- plants from each line are half-sibs, sharing the same maternal lineage, but not necessarily the same paternal lineage.

Seedlings of *F. rubra* (E+, E−) from lines PEN and RAB were grown in a mixture of peat moss and vermiculite (2:1 v/v) in a glasshouse. The mixture had pH 5.3; 70.2% organic matter; 7.6 g kg^-1^ total nitrogen and 256 mg kg^-1^ available phosphorus. After four months, the presence of *Epichloë festucae* in plants was verified by isolation of the fungus from leaf sheaths on PDA, as previously indicated. The E- status of treated plants was also verified in the same way. Two months later, plants of similar size with two tillers were selected. Each plant was weighted after trimming to 5-6 cm of aboveground height and 5 cm of roots. Plants with similar fresh weight were selected and individually transplanted to 12 cm diameter pots containing the potting mix and kept in a glasshouse during eight weeks with regular watering.

### Experimental design

The experiment lasted six weeks. Before the water treatments started, soil in all pots was fully saturated with tap water. For the next two weeks two water treatments were imposed: one third of the plants were watered on alternate days to field capacity (C= control); and water was withheld in the remaining two thirds of the plants (W1 and W2 treatments). After this period, treated plants had visible drought symptoms like leaf rolling and colour changes, and then they were watered on alternate days to field capacity during two weeks. A second drought period was then imposed by withholding water during 7 days (W1= moderate stress) or 14 days (W2= severe stress). After this second-drought period, the mean water content of W1 and W2 pots was 51% and 32% of their field capacity, respectively. Two drought treatments were chosen because, in dehesa grasslands rainfall is irregularly distributed during the period of vegetative growth (spring and autumn), and plants usually experience several periods of drought.

The experiment consisted of a three-way factorial arrangement within a randomised design with six replications of plant line (PEN and RAB), endophyte infection status (E+ and E−) and water treatment (C, W1 and W2). The experiment contained a total of 72 pots which were placed at random in a glasshouse at a temperature regime of 22°C day and 15°C night, and ambient light conditions of late spring. During the experiment, the pots were shuffled every four days to avoid position effects. After the six weeks of the drought treatment the plants were harvested, splitting roots and shoots (green leaves and dry leaves). Water content in shoots was determined at harvest. Shoot and root dry matter was determined in freeze-dried plant material. 

To observe if mycorrhizal fungi were associated to the roots of fescue plants, samples of a few roots of two E+ and E- plants of each line and water treatment were stained with tryphan blue and observed by microscopy [56]. Roots of *F. rubra* plants which have been growing in a field research plot for two years were used as controls growing in a natural soil.

### Chemical analyses

The concentration of mineral elements was determined in root and shoot tissues. After combustion of dried and ground plant samples at 450°C for 8 hours, ashes were dissolved in an acid mixture [57]. Then, phosphorus concentration was determined colorimetrically as molybdo-vanado-phosphoric acid using a Cary 100 Conc Spectrophotometer. The K, Ca, Mg, and Zn concentrations were determined by atomic absorption spectrophotometry. Nitrogen was analyzed using the Kjeldahl distillation method [58]. 

Proline was quantified in root and shoot tissues using the spectrophotometric method described by Bates [59]. Approximately 50 mg of freeze dried plant material were homogenized in 1 ml of 3% aqueous sulfosalicylic acid and kept for 10 minutes in ice. The mixture was centrifuged and 500 μl of the supernatant were mixed with 1 ml of acid ninhydrin and 500 μl of glacial acetic acid in a Pyrex tube. The mixture was heated for 1 h at 90°C, and after it cooled it was extracted with 2 ml of toluene. The chromophore-containing toluene was discarded, and the absorbance was measured at 520 nm in a Cary 50 Probe Spectrophotometer. Standards of L(−) proline (Acrós Organics) were used for quantification of proline in the samples.

The concentration of total phenolic compounds (TPhC) was estimated in extracts of root and shoot tissues. For each plant sample, the extraction procedure was performed in duplicate as follows. A 200 mg aliquot of each freeze-dried and ground plant sample was extracted twice in 5.0 ml of 70:30 ethanol:water (v/v) for 30 min in an ultrasound bath. The mixture was centrifuged and filtered twice through filter paper. The TPhC concentration of plant extracts was determined by colorimetry using Folin-Ciocalteu reagent [60]. A 150 μl aliquot of each sample was mixed with 3 ml of distilled water followed by addition of 250 μl of Folin-Ciocalteu reagent (Scharlab Chemie S.A.). After 6 minutes, 750 μl of a 7% Na_2_CO_3_ solution were added and absorbance was measured after 120 min, at 760 nm in a Cary 50 Probe Spectrophometer, using gallic acid (Acrós Organics) as a reference standard for quantification.

 The content of two fungal alkaloids, ergovaline and peramine, was analyzed in aboveground plant tissue samples. For this, three replicates of each treatment, endophyte and plant line combination (a total of 36 samples) were considered. The levels of ergovaline and its isomer ergovalinine were quantified by HPLC following a modification of the methods described by Hill [61] and Yue [62]. A 1.0 g shoot sample was extracted in 20 ml of CHCl_3_ and 1 ml of 0.5 mM NaOH for 2 hours. One hundred microliters of an internal standard solution of ergotamine ditartrate (10 μg ml^-1^, Sigma-Aldrich) were added to the sample prior to extraction. The mixture was vacuum-filtered through Whatman n° 2 filter paper and a 10 ml aliquot of filtrate was passed through a 500 mg Ergosil (Analtech; Newark, USA) solid-phase column preconditioned with CHCl_3_. Plant pigments were removed with 5 ml of chloroform:acetone (1:3). The sample was eluted with 2 ml of methanol and vacuum concentrated, redissolved in 1 ml of methanol, and filtered through a 0.22 μm nylon filter. Extracts were chromatographed with a Waters 2690 system with an Xterra MS C18 Waters column (4.6 × 100 mm) and a guard column (3.9 × 20 mm) of the same characteristics. The initial solvent gradient was 35% acetonitrile in 0.01 M ammonium acetate buffer (pH = 7.6), with a flow rate of 0.8 ml min^-1^. The gradient was adjusted through time programming as follows: step 1, 35% to 50% acetonitrile in a 20 min linear gradient; step 2, 50% for 5 min; step 3, 50% to 90% in a 5 min linear gradient; step 4, 90% to 35% in a 5 min linear gradient. Ergovaline and its isomer were detected by fluorescence spectrophotometry with an excitation wavelength of 250 nm and an emission wavelength of 420 nm (Waters Fluorescent Detector 2475). Ergovaline and ergovalinine areas were combined, and the total was reported as ergovaline. A standard was prepared by adding ergovaline (provided by Forrest Smith, Auburn University, USA) to a 1.0 g sample of a non-infected red fescue plant free of ergovaline, which was treated as described above. The limit of detection was 0.01 mg kg^-1^.

Peramine was determined using the HPLC method described by Barker [63] and Yue [62]. A freeze-dried and ground sample (100 mg) was extracted in 3 ml of 30% isopropanol for 30 min at 90°C. The mixture was centrifuged and the extract was passed through a precondioned Varian Bond Elut carboxylic acid (CBA) column packed with 100 mg of adsorbent. After a wash of the column with 1-2 ml of methanol, peramine was eluted with 1 ml of 5% formic acid in 80% aqueous methanol. The extract was filtered through a 0.22 μm nylon filter and chromatographed in a Waters 2690 system with a Nova Pak C18 Waters column (3.9 × 150 mm). The isocratic mobile phase consisted of 18% (v/v) acetonitrile in a guanidine carbonate (10 mM)-formic acid buffer. Detection was performed with a Photodiode Array Detector (PDA) Waters 2996 set at 280 nm. The peramine standard was a gift from Geoffrey Lane (AgResearch, New Zealand). The limit of detection was 0.8 μg g^-1^.

### Statistical analyses

Effects of plant line, endophyte infection status and water treatment on biomass production and chemical composition were analysed with a three-way ANOVA. Differences between estimated effects for fixed factors and their interactions were assessed using the least significant difference (LSD) at *P* = 0.05. These data analyses were performed with IBM SPSS (Statistics 19).

The HJ-biplot representation technique allowed us to analyze all variables simultaneously. This technique achieves an optimum quality of representation for both rows (samples) and columns (variables) of a data matrix, as both are represented on the same reference system [64]. The method is closely related to main component analysis, as covariance matrices are plotted on planes which account for most of the inertia. Short distances between row points are interpreted in terms of similarity and long distances as dissimilarities. The angle formed by two vectors (variables) is interpreted as a correlation. The smaller the angle between two vectors or variables, the greater is their correlation; a 90° angle is interpreted as independence, and a 180° angle means an inverse correlation. If a row point (sample) is close to a column point (variable) or to its prolongation, it is interpreted as preponderance. On projecting all row markers perpendicularly onto the directions of any of the variables, the order of the projections in the direction of those variables is equivalent to the value taken on the variable. Several measures are essential for a correct HJ-Biplot interpretation [65]. Thus, the relative contribution of the factor to the element (CRFE) expresses the part of the variability of the element (row or column) explained by the factor (axis). The quality of representation is the sum of the relative contribution of the factors considered. Only the points with good quality of representation can be interpreted correctly in the subspace observed. A quality of representation is considered acceptable when its value is above 500 in a 0-1000 scale. 

The inertia criterion based on a HJ-Biplot representation [66,67] starts from the configuration of the resulting information obtained in the HJ-Biplot, which retains an adequate number of axes. Based on this configuration we obtained a hierarchical ascendant classification whose classes are easily interpretable, in which we know the variables responsible for the classification of different representations obtained by the HJ-biplot. For the analysis we considered a matrix of 72 rows (samples) and 19 columns (variables) to search for clusters (associations of samples). The rows refer to samples considering the three factors: plant line (PEN, RAB), endophyte status (E+, E−), and water treatment (C, W1, W2) in a factorial combination with six replicates. The variables considered were: shoot biomass (DW_shoot_), root biomass (DW_root_), dead leaves (DE_shoot_), the mineral element concentration N_shoot_, N_root_, P_shoot_, P_root_, K_shoot_, K_root_, Ca_shoot_, Ca_root_, Mg_shoot_, Mg_root_, Zn_shoot_, Zn_root_, and the secondary metabolites Proline_shoot_, Proline_root_, TPhC_shoot_, TPhC_root_. Multbiplot software was used for this statistical analysis [68].

## Results

### Shoot and root biomass

**Figure 1:**
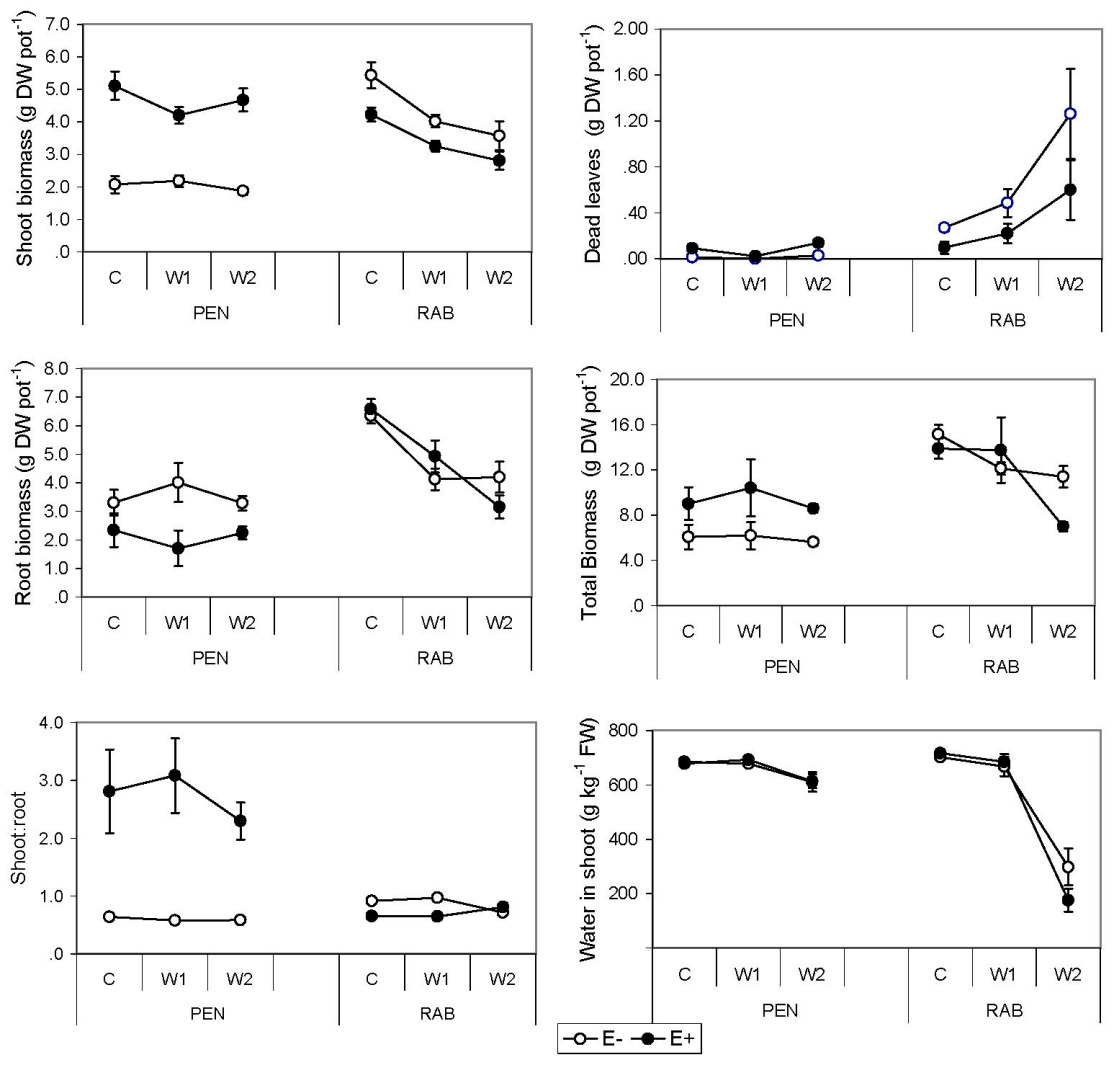
Biomass production and water content of *Festuca* *rubra* plants. Effect of fungal endophyte status (• = E+, infected; ο = E-, non-infected) of two lines (PEN and RAB) of plants, growing at different water treatments (C= control; W1= moderate stress; W2= severe stress). Values, expressed as g of dry weight (DW) per pot, are means ± 2 SE (n=6).

The endophyte status had a significant effect on shoot and root biomass and dead leaves, dependent on the plant line (line × endophyte, Table 1). In the PEN line E+ plants had greater shoot biomass than E- plants; but the opposite occurred in the RAB line (Figure 1). Root biomass of the PEN line was greater in E- than E+ plants, and in the RAB line differences were not statistically significant. The RAB plants had more dry leaves than the PEN plants, and in the RAB line, E- plants had more dry leaves than E+ plants (Figure 1). Total biomass and water content in aboveground tissues were not significantly affected by endophyte infection status or its interaction with other factors. Both plant lines showed important differences in biomass allocation as affected by the interaction with the endophyte status (line × endophyte, Table 1). In the PEN line, the endophyte increased the S:R ratio more than three times. This was due to E+ plants having greater shoot and lower root biomass than E- (Figure 1). Thus, with smaller roots, E+ plants produced more shoot biomass than E- plants. In the RAB line, the ratio was only slightly greater in E- than in E+ plants.

**Table 1:** Summary of ANOVA results for the effect of plant line, endophyte status and water treatment on growth of *Festuca rubra*.

		**Shoot biomass**		**Root biomass**			**Shoot:Root**		**Dead leaves**		**Total biomass**		**Shoot water**	
**Effect**	**df**	***F***	***P***	***F***	***P***	***F***		***P***	***F***	***P***	***F***	***P***	***F***	***P***
Line (L)	1	9.75	**0.003**	59.4	**0.000**	30.8		**0.000**	27.8	**0.000**	15.9	**0.000**	46.9	**0.000**
Endophyte (E)	1	24.8	**0.000**	7.15	**0.011**	38.1		**0.000**	3.14	0.081	0.05	0.821	0.57	0.452
Treatment (T)	2	12.5	**0.000**	9.98	**0.000**	0.668		0.522	8.33	**0.001**	5.60	**0.008**	103.3	**0.000**
L x E	1	107.6	**0.000**	7.13	**0.011**	52.0		**0.000**	6.89	**0.011**	2.75	0.106	0.94	0.336
L x T	2	5.11	**0.009**	9.42	**0.001**	0.433		0.652	6.48	**0.003**	2.89	0.068	55.1	**0.000**
E x T	2	0.47	0.627	0.549	0.583	0.122		0.885	0.64	0.527	0.25	0.776	1.90	0.158
L x E x T	2	1.55	0.220	2.80	0.074	1.241		0.301	1.01	0.367	2.22	0.123	1.75	0.182

Significant *P*-values are in bold (*P* < 0.05)

With decreasing water availability there was a significant decrease in shoot and root biomass in the RAB line, although differences between W1 and W2 treatments were not statistically significant (line × treatment, Table 1; Figure 1). However, differences between water treatments were not statistically significant in the PEN line. In agreement with this, there were no significant changes in the amount of dead leaves across treatments in the PEN line, but in the RAB line dead leaves significantly increased with drought (Figure 1). Total biomass was not significantly affected by the line × treatment interaction (Table 1). Water content in aboveground tissue varied with the interaction between plant line and water treatment; thus, in the RAB line there was a decrease in water content of tissues with increasing drought, but in the PEN line differences between treatments were not statistically significant (Figure 1). 

When roots of the experimental E+ and E- plants were stained and observed at the microscope, no root-associated fungi were observed. However, hyphae associated to the root epidermis and cortex were common in the roots of plants obtained from a field plot. 

### Mineral concentration

**Figure 2:**
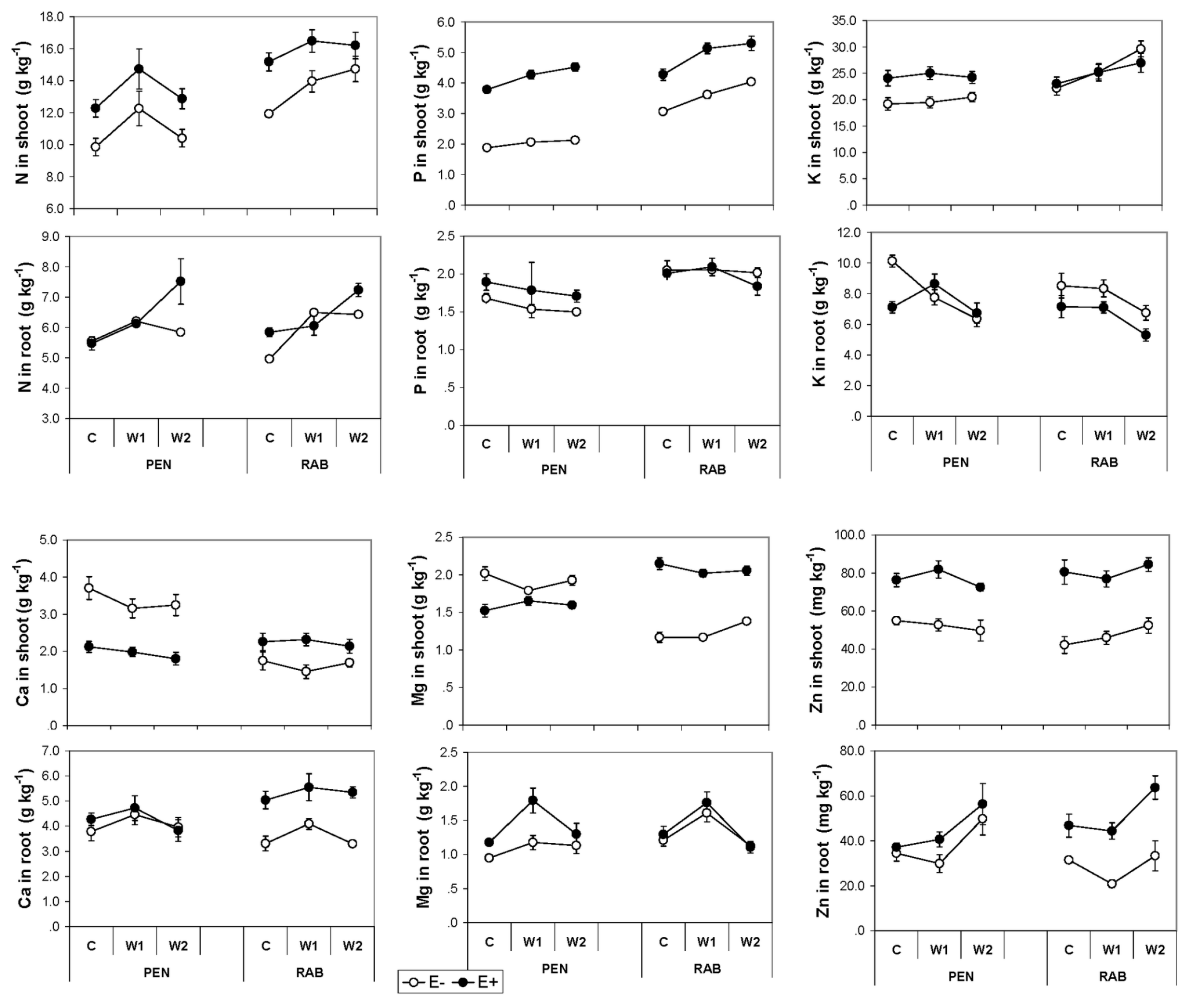
Nutrient concentration of *Festuca* *rubra* plants. Effect of fungal endophyte status (• = E+, infected; ο = E-, non-infected) of two lines (PEN and RAB) of plants, growing at different water treatments (C= control; W1= moderate stress; W2= severe stress). Values are means ± 2 SE (n=6).

The concentration in shoots of all mineral elements measured was significantly affected by the endophyte status of the plant (Table 2). Infected plants had greater concentrations of N and Zn in shoots than E- plants (Figure 2). In spite of the line × endophyte interaction, in both plant lines E+ plants had greater P in their shoots than E- plants (Figure 2). The effect of the endophyte on the concentrations of K, Ca and Mg in shoots depended on the plant line (line × endophyte, Table 2). Thus, the contents of Ca and Mg in the RAB line were greater in E+ than in E- plants, but in the PEN line they were greater in E- plants (Figure 2). The K concentration of PEN was greater in E+ than in E- plants, but differences between RAB plants were not statistically significant.

**Table 2:** Summary of ANOVA results showing the level of significance for the effect of plant line, endophyte status and water treatment on chemical composition of *Festuca rubra* in shoots.

		**N shoot**		**P shoot**		**K shoot**		**Ca shoot**		**Mg shoot**		**Zn shoot**		**Proline shoot**		**TPhC shoot**	
**Effect**	**df**	***F***	***P***	***F***	***P***	***F***	***P***	***F***	***P***	***F***	***P***	***F***	***P***	***F***	***P***	***F***	***P***
Line (L)	1	35.8	**0.000**	222	**0.000**	18.2	**0.000**	36.6	**0.000**	6.79	**0.012**	0.16	0.685	83.1	**0.000**	6.10	**0.018**
Endophyte (E)	1	29.4	**0.000**	531	**0.000**	6.95	**0.011**	10.6	**0.002**	51.6	**0.000**	152	**0.000**	0.11	0.734	2.09	0.156
Treatment (T)	2	7.48	**0.002**	33.8	**0.000**	5.74	**0.005**	1.62	0.205	1.85	0.166	0.10	0.900	112	**0.000**	0.06	0.934
L x E	1	0.00	0.968	30.8	**0.000**	11.9	**0.001**	68.3	**0.000**	262	**0.000**	3.91	0.052	1.22	0.272	4.19	**0.048**
L x T	2	1.78	0.186	3.97	**0.024**	3.46	**0.038**	0.56	0.572	0.53	0.590	3.08	0.053	68.3	**0.000**	1.02	0.370
E x T	2	0.31	0.736	1.54	0.221	0.94	0.394	1.00	0.372	2.32	0.107	0.12	0.880	1.03	0.360	0.02	0.973
L x E x T	2	0.35	0.707	0.90	0.411	0.20	0.818	0.06	0.940	5.01	**0.010**	0.87	0.424	2.04	0.138	0.51	0.602

Significant *P*-values are in bold (*P* < 0.05)

TPhC = total phenolic content

The water treatment had a significant effect on the concentrations of N, P and K in shoots (Table 2). Phosphorus significantly increased with decreasing water availability (Figure 2). Nitrogen increased from C to W1, but no differences between W1 and W2 treatments were found; and the K concentration increased from W1 to W2. The concentrations of Ca, Mg and Zn in shoots were not significantly affected by the water treatment (Table 2).

In spite of the line × endophyte interaction, in both plant lines, E+ roots had greater Ca, Mg and Zn, and lower K concentration than E- roots (Table 3; Figure 2). The endophyte status did not affect the N and P in roots. The water treatment affected the N, K, Ca, Mg and Zn concentrations in roots, but did not have a significant effect on their P concentration. In general, with increasing drought the N and Zn concentrations tended to increase and the K to decrease (Figure 2). Nitrogen increased in the W1 treatment, but differences between W1 and W2 were not statistically significant. The highest Ca and Mg concentrations were found in the W1 treatment, but differences between C and W2 were not statistically significant for these nutrients. 

**Table 3:** Summary of ANOVA results showing the level of significance for the effect of plant line, endophyte status and water treatment on chemical composition of *Festuca rubra* in roots.

		**N root**		**P root**		**K root**		**Ca root**		**Mg root**		**Zn root**		**Proline root**		**TPhC root**	
**Effect**	**df**	***F***	***P***	***F***	***P***	***F***	***P***	***F***	***P***	***F***	***P***	***F***	***P***	***F***	***P***	***F***	***P***
Line (L)	1	0.02	0.873	18.2	**0.000**	3.53	0.065	1.71	0.196	2.21	0.142	0.21	0.644	13.1	**0.003**	300	**0.000**
Endophyte (E)	1	2.16	0.156	1.12	0.292	9.34	**0.003**	23.0	**0.000**	9.61	**0.003**	28.2	**0.000**	4.04	0.067	41.4	**0.000**
Treatment (T)	2	6.52	**0.006**	1.24	0.296	14.9	**0.000**	3.84	**0.027**	18.0	**0.000**	13.3	**0.000**	21.0	**0.000**	16.2	**0.000**
Lx E	1	0.02	0.886	3.51	0.066	1.49	0.226	14.2	**0.000**	4.13	**0.046**	8.61	**0.005**	6.46	**0.026**	23.9	**0.008**
L x T	2	0.07	0.930	0.42	0.659	0.08	0.918	0.16	0.852	2.22	0.117	0.73	0.482	15.3	**0.000**	7.89	0.349
E x T	2	1.92	0.171	0.24	0.782	3.85	**0.027**	0.13	0.878	1.94	0.153	1.12	0.332	3.56	0.061	1.08	**0.049**
L x E x T	2	0.83	0.448	0.11	0.893	3.70	**0.031**	0.62	0.542	0.58	0.561	0.43	0.652	7.02	**0.010**	3.28	0.488

Significant *P*-values are in bold (*P* < 0.05)

TPhC = total phenolic conten

### Proline and secondary metabolites

**Figure 3:**
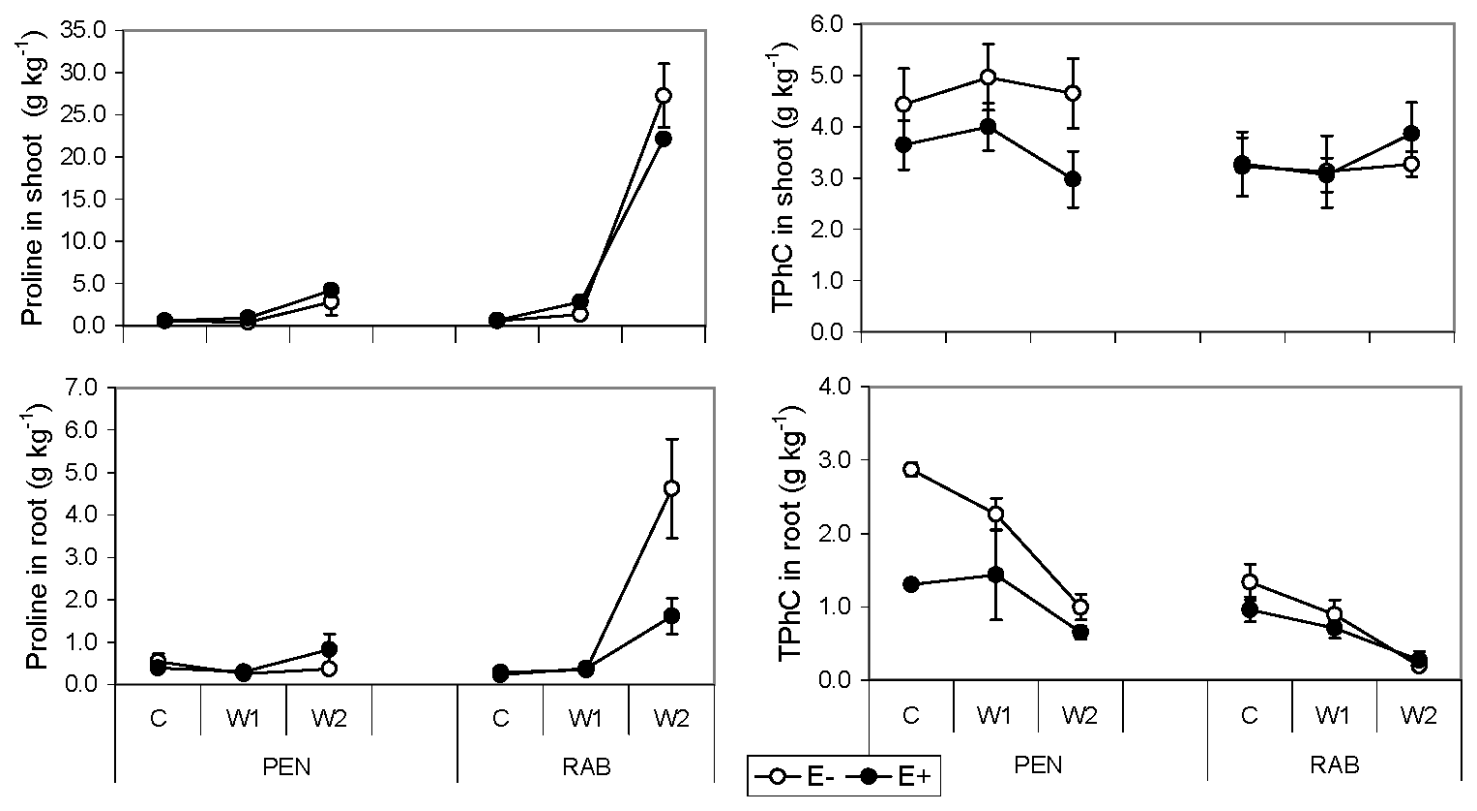
Proline and total phenolic compounds (TPhC) of *Festuca* *rubra* plants. Effect of fungal endophyte status (• = E+, infected; ο = E-, non-infected) of two lines (PEN and RAB) of plants, growing at different water availability levels (C= control; W1= moderate stress; W2= severe stress). Values are means ± 2 SE (n=6).

The endophyte status did not affect the proline content in shoots (Table 2). However, in roots there was a significant line × endophyte × treatment interaction (Table 3), and in the RAB line, E+ plants had much lower proline than E- at the W2 treatment (Figure 3). Proline in shoots and roots increased with decreasing water availability, although differences between C and W1 treatments were not statistically significant. The increase in proline in shoot and roots of RAB plants at the W2 treatment was up to five times greater than in PEN plants. 

The concentration of total phenolic compounds (TPhC) in shoots was significantly affected by endophyte status depending on plant line (line × endophyte, Table 2). Thus, E- plants of PEN line had a greater concentration than E+, but in RAB line differences were not significant (Figure 3). In both plant lines, roots of E- plants had a significantly greater TPhC concentration than E+. The TPhC in roots decreased with decreasing water availability (Table 3, Figure 3). 

**Figure 4:**
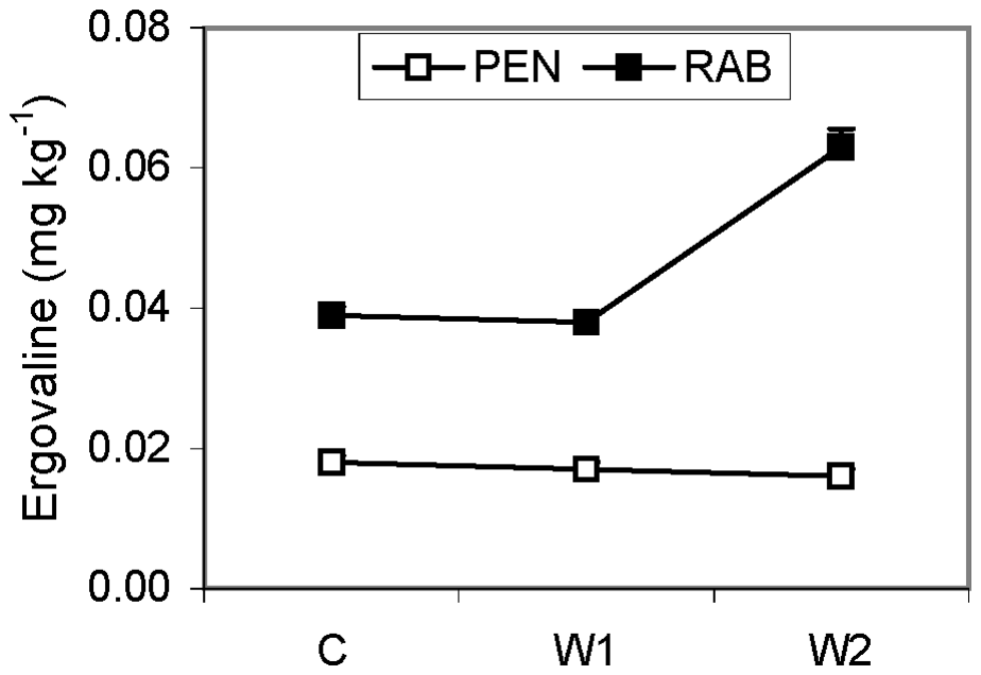
Ergovaline concentration in endophyte infected (E+) plants. Two lines (☐= PEN; ■= RAB) of plants, growing at different water treatments (C= control; W1= moderate stress; W2= severe stress). Values are means ± 2 SE (n=6).

The infected plants of RAB line had a greater ergovaline concentration than those of the PEN line, particularly in the W2 treatment (Figure 4). Peramine was not detected in any sample. Non-infected plants did not have detectable amounts of peramine or ergovaline alkaloids.

### Classification by means of inertia criterion based on HJ-biplot

**Figure 5:**
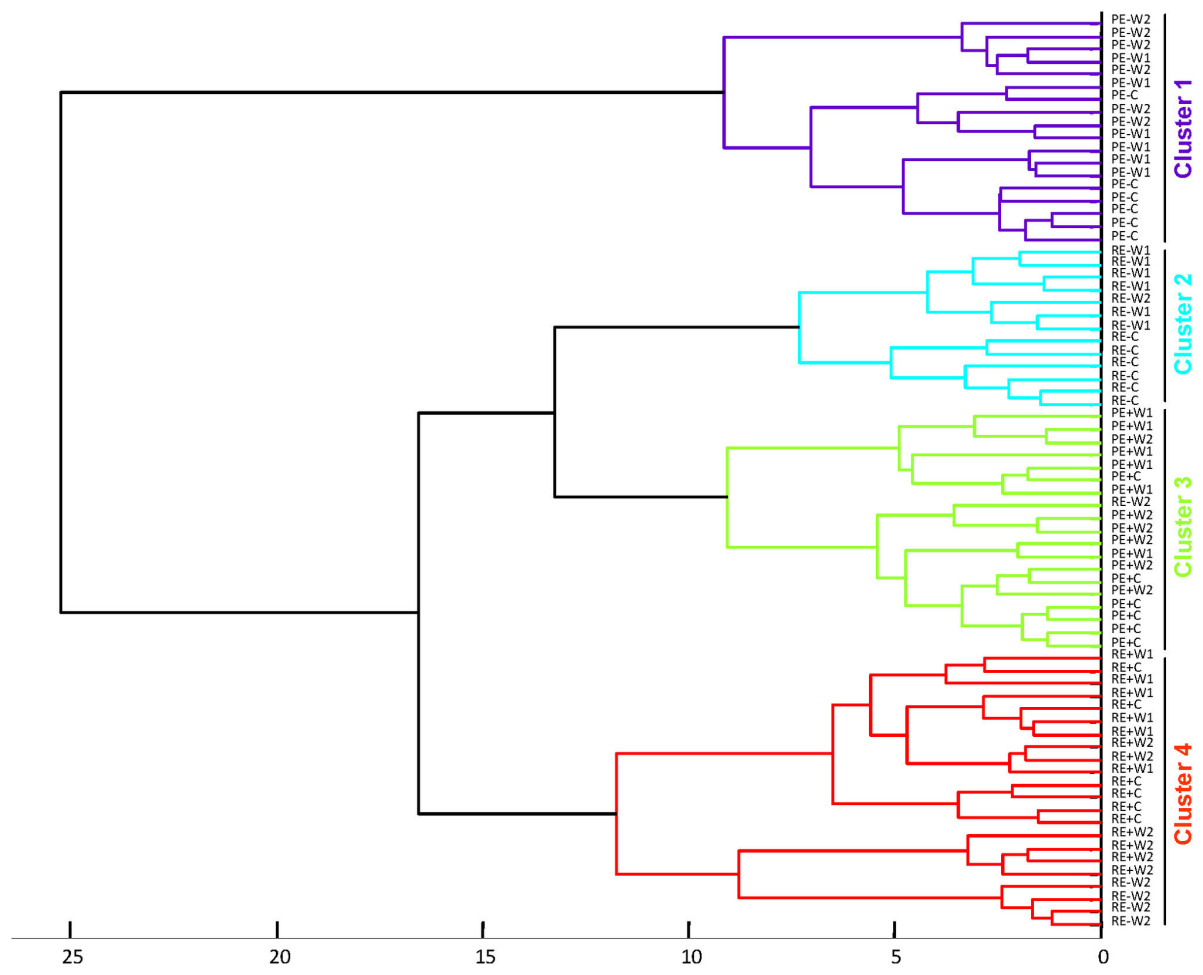
Hierarchical cluster with the Euclidean distance using the biplot scores. Each sample was labelled with a first letter for plant line (P= PEN; R= RAB), a second letter for endophyte status (E+= endophyte infected; E-= non-infected), and a third letter for water treatment (C= control; W1= moderate stress; W2= severe stress). The cophenetic correlation coefficient was 0.679.

**Figure 6:**
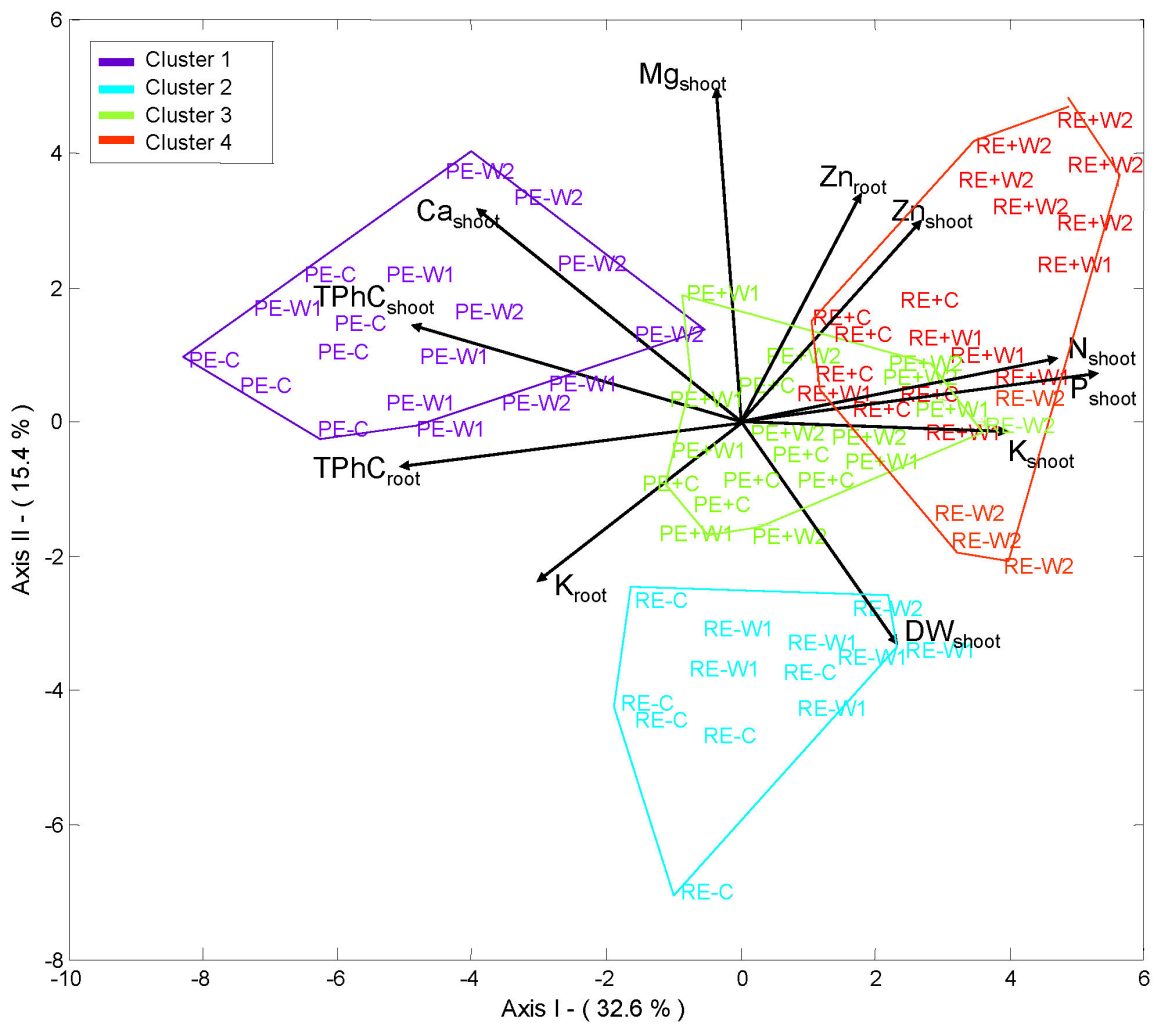
H-J biplot representation of samples and variables on the factorial plane I-II. Each sample was labelled with a first letter for plant line (P= PEN; R= RAB), a second letter for endophyte status (E+= endophyte infected; E-= non-infected), and a third letter for water treatment (C= control; W1= moderate stress; W2= severe stress). Variable labels: DW_shoot_ = shoot biomass; DW_root_=root biomass; TPhC_shoot_= total phenolic compounds in shoots; TPhC_root_= total phenolic compounds in roots; Proline_shoot_= proline in shoots; Proline_root_= proline in roots; and nutrient concentration in shoot and roots: N_shoot_, N_root_, P_shoot_, P_root_, K_shoot_, K_root_, Ca_shoot_, Ca_root_, Mg_shoot_, Mg_root_, Zn_shoot_, Zn_root_.

Using the HJ-biplot method on the matrix containing the original data, retained by seven axes, an 83.3% of the variance was explained. Hierarchical clustering of samples clearly distinguished four groups, according to the interaction between the endophyte status (E+, E−) and plant line (PEN, RAB), but not related to the water treatment (C, W1, W2) (Figure 5). In order to determine which variables were responsible of this segregation, each cluster was analyzed separately. The four clusters were analyzed on the factorial planes I-II and I-III (Figures 6, S1). Only variables with acceptable quality representation could be interpreted correctly and therefore they were included in the factorial planes.

Cluster 1 contained all non-infected samples from PEN line (PE−), only these samples occurred in this cluster. Most samples of this cluster had a high quality of representation on the factorial plane I-II. Samples were located on the negative part of axis I, characterized by high values in Ca_shoot_, TPhC_shoot_, TPhC_root_, and low values in P_shoot_, N_shoot_ and K_shoot_, three highly correlated variables on the positive part of the axis I (Figure 6). 

Non-infected plant samples from RAB line (RE−) made up a second cluster, but not all RE- samples were included here; four RE- samples were included in the cluster 4. The cluster 2 had its best quality of representation on the plane I-II (Figure 6); and samples were on the negative part of axis II and characterized by high values in DW_shoot_ and K_root_, and low values in Mg_shoot_, Ca_shoot_ and Zn_root_. 

In the cluster 3 all infected plant samples from line PEN (PE+) and one non-infected of line RAB (RE−W2) were gathered. This sample set was located in the centre of the plane I-II showing low variability on most of variables (Figure 6). The quality of representation in this plane was low; however, most samples were well represented on the factorial plane I-III (Figure S1). Axis III discriminated samples along a decreasing gradient in Mg_root_ and Ca_root_, and increasing in Proline_shoot_: on the positive part W1 treatment samples with high contents in Mg_root_ and Ca_root_ were located, and on the negative part there were samples with high values in Proline_shoot_, the intermediate part was occupied by C treatment samples which had low variability on these variables. 

Finally, in the cluster 4 all infected plant samples from the RAB line (RE+) and four RE−W2 samples were gathered. Most of them had high quality of representation on the factorial plane I-II and high values for the variables Zn_shoot_, Zn_root_, N_shoot_, P_shoot_, K_shoot_ and low values for the variables Ca_shoot_, TPhC_shoot_ and TPhC_root_ (Figure 6). Within this cluster a subset formed by RE+W2 samples was differentiated from RE+W1 and RE+C samples. This segregation was established due to decreasing values in N_shoot_, P_shoot_, Zn_shoot_, Zn_root_ with increasing water availability in samples, from treatment W2 to treatment C. Samples RE−W2 were clustered in another subset, these samples were well represented in the plane I-III and were related to Proline_shoot_ (Figure S1).

## Discussion

The HJ-biplot analysis allowed a comprehensive analysis of all variables (including biomass production, nutrient content, proline and secondary metabolites of both aboveground and belowground tissues), and showed a clear distinction of four clusters corresponding to the plant lines and their endophyte infection status. These results indicate that endophyte status and plant line imposed stronger differences in the performance of *F. rubra* plants than the water stress treatments. Furthermore, differences between PEN and RAB lines seemed to be greater in E- plants than in E+ plants (Figure 6), this suggests that infected plants of both lines are more similar than those of their non-infected versions. This might be due to the endophyte producing a similar effect in both plant lines, by increasing the concentration of N, P and Zn in shoots. Thus, the clusters formed by PE+ and RE+ samples were close to each other and related to the variables P_shoot_, N_shoot_, Zn_shoot_ and Zn_root_ (Figure 6). On the other hand, the RE- samples were separated from PE- and more strongly related to high DW_shoot_. It is important to point out that the observed increase in nutrient concentration due to the endophyte was not dependent on the *F. rubra* host plant line. Endophyte effects on plant performance are either elusive or reported to be determined by host plant genotype [17,20–23,25,28,33,35]. Our results were based on only two *F. rubra* plant lines, but it is remarkable that in spite of the large differences between lines in shoot and root biomass, the effect of fungal endophyte was similar for the nutrients P, N and Zn, as shown by the sample ordination (Figure 6). 

Our results strongly suggest that the endophyte modifies the nutrient balance in *F. rubra*: in spite of differences in biomass production and allocation between plant lines, the endophyte increased the concentration of P, N and Zn in shoots, and of Ca, Mg, and Zn in roots, and this occurred in both plant lines and all water treatments. On average, infected plants contained in their shoots more P (62%), Zn (58%) and N (19%) than non-infected plants. A positive effect of an endophyte on total aminoacid concentrations was also reported in a single cultivar of tall fescue [69]; however, the N concentration was not affected by the endophyte in *F. rubra* [70], and it was significantly lower in infected plants of *Achnatherum sibiricum* [71]. The greater Zn content in shoots and roots contrasts with the observations of Malinowski [22] and Monnet [21] who found that *Neotyphodium* endophytes did not affect the Zn concentration in tall fescue or ryegrass shoots.

Our results showed an endophyte-mediated increase in aboveground P, in both plant lines. On the contrary, Li [71] did not find differences in P concentration between shoots of E+ and E- plants of *Achnaterum sibiricum* under different N-P soil availability levels. Malinowski [41] found a greater P concentration in endophyte-infected tall fescue, but only when the soil P was low, suggesting that this benefit of endophyte symbiosis only occurs under low P availability. By contrast, in our study the P concentration of aboveground plant parts was greater in E+ plants of both lines in a substrate rich in P (256 mg kg^-1^ soil) and at all levels of water stress (Figure 2). On average infected plants contained 62% more P than E- plants. Such a P increase was greater than the one we found in a previous field experiment with five lines of *F. rubra* growing in a soil with low P availability [72]. This suggests that *Epichloë festucae* can increase the P concentration of *F. rubra* plants independently of water availability, plant line, and probably P soil content. This is a relevant finding contrasting with Malinowski [41] who found endophyte benefit to be dependant on P availability. The chemical configuration of this additional P found in E+ plants is not known and would be an interesting subject for further research. 

A variety of strategies are used by plants to mobilize and acquire P from the soil [73]. Many plants foster symbiotic relationships with mycorrhizal fungi to increase P absorption; however, we did not observe the presence of any root-associated fungi in the E+ or E- *F. rubra* plants. Plant roots acidify the rhizosphere and secrete low molecular weight organic anions (ie. phenolic compounds, organic acids) and phosphatase enzymes into the soil to mobilize P [73]. Although epichloid endophytes only colonise aerial plant tissues and are not present in roots, it is clear that root metabolism could be affected by endophyte infection. For instance, Malinowski [74] observed an increase in root exudation of phenolic compounds in tall fescue associated to *Neotyphodium coenophialum*. In our experiment we did not find differences in total phenolic compounds between roots of E+ and E- plants, and did not measure exudated phenolics (Figure 3), however, other types of exudates could be involved in P mobilization in E+ plants. For example, Vázquez de Aldana [75] found that *Epichloë* infection increased the competitive ability of red fescue plants due to root-mediated allelopathic interactions, and Li [71] detected a greater acid phosphatase secretion by roots in E+ plants of *Achnatherum sibiricum*.

We found clear differences between plant lines, irrespective of endophyte infection, in response to the water treatment. The two plant lines originated from *F. rubra* plants collected at two different locations 74 km apart that differ in the type of soil substrate (La Peña on granite, Raboso on sediments), but have similar annual precipitation values. Our results showed that under the conditions of our experiment the plants of the PEN line were drought tolerant, being not significantly affected by water stress in terms of root or shoot biomass, amount of dead leaves, water content in shoots, or proline content (Figures 1,3). On the contrary, the RAB line was drought sensitive, showing a decrease in shoot and root biomass and shoot water content, and an increment of dead leaves and proline content. Although infected PEN plants had smaller root biomass than all other plants analyzed, they produced the greatest shoot biomass under drought. The above results indicate that widely different growth responses to drought occur among *F. rubra* germplasm from dehesa grasslands. Furthermore, endophytes can affect these growth responses. To maintain growth under drought is not necessarily an indication that the plant is drought tolerant. For the persistence of grasses in semiarid environments, survival and recovery after drought might be inversely correlated with growth under drought [76]. That is, the most important strategy is not the biomass production during drought, but the ability to survive and recover rapidly after the dry season. 

Accumulation of the osmotically active metabolite proline in plant tissues is correlated with drought resistance in many crops [77]. While proline levels in shoots increased in response to water stress, the *Epichloë* endophyte did not affect this response (Figure 3). Similar results were reported in endophyte-infected *Lolium perenne* [36]. On the other hand, the greater proline concentration in roots of E- RAB plants compared to E+ plants at the lowest water availability and the larger amount of dead leaves suggests that E+ plants were less stressed, which agrees with results reported for endophytic tall fescue shoots [17,23]. Proline metabolism may be affected in E+ plants because this amino acid is a product of ergot alkaloid breakdown and peramine synthesis, both produced by the endophyte [16,78]. The peramine alkaloid was not detected in any sample, but the levels of ergovaline increased in response to increasing water stress in the plant line most sensitive to drought (RAB) (Figure 4), and correlated with an increase of proline in shoots and roots (Figure 3). A similar ergovaline increase in response to drought was reported in tall fescue [44] and ryegrass [36]. This suggests that like proline, ergovaline might accumulate in certain plant genotypes, in response to water stress. 

There is increasing evidence that alterations in the expression of antioxidant compounds (ie. phenolics), as a result of endophyte infection could be a mechanism by which the endophyte symbiosis enhances the resistance of grass hosts to multiple stress factors [79-81]. We did not observe an accumulation of TPhC with drought in any plant; however, differences in TPhC occurred between plant lines, with a greater content in PEN, the plant line less affected by drought.

In conclusion, our results showed that the endophyte *Epichloë festucae* did not increase the resistance of *Festuca rubra* plants to drought. Instead, differences in biomass production and proline content in response to water availability occurred between plant lines. However, we found a clear effect of the endophyte on plant nutrition, irrespective of plant line and water treatment. Infected plants had a significant increase in N, P and Zn in their shoot tissues, and Zn in roots. Effects of endophyte infection on plant nutrition could explain the high prevalence of infected plants observed in natural grasslands. The results of this study also suggest that nutrient incorporation characteristics of *F. rubra* cultivars could be improved using adequate *Epichloë* endophytes. 

## Supporting Information

Figure S1 **H-J biplot representation of samples and variables on the factorial plane I-III.** Each sample was labelled with a first letter for plant line (P= PEN; R= RAB), a second letter for endophyte status (E+= endophyte infected; E-= non-infected), and a third letter for water treatment (C= control; W1= moderate stress; W2= severe stress). Variable labels: DW_shoot_ = shoot biomass; DM_root_=root biomass; TPhC_shoot_= total phenolic compounds in shoots; TPhC_root_= total phenolic compounds in roots; Proline_shoot_= proline in shoots; Proline_root_= proline in roots; and nutrient concentration in shoot and roots: N_shoot_, N_root_, P_shoot_, P_root_, K_shoot_, K_root_, Ca_shoot_, Ca_root_, Mg_shoot_, Mg_root_, Zn_shoot_, Zn_root_.
(TIF)
