# Supplementary figures and images for: Fungal Endophyte (Epichloë festucae) Alters the Nutrient Content of Festuca rubra Regardless of Water Availability
Source: PLoS One. 2013 Dec 18;8(12):e84539. doi: 10.1371/journal.pone.0084539 (PMC3867530; doi:10.1371/journal.pone.0084539)

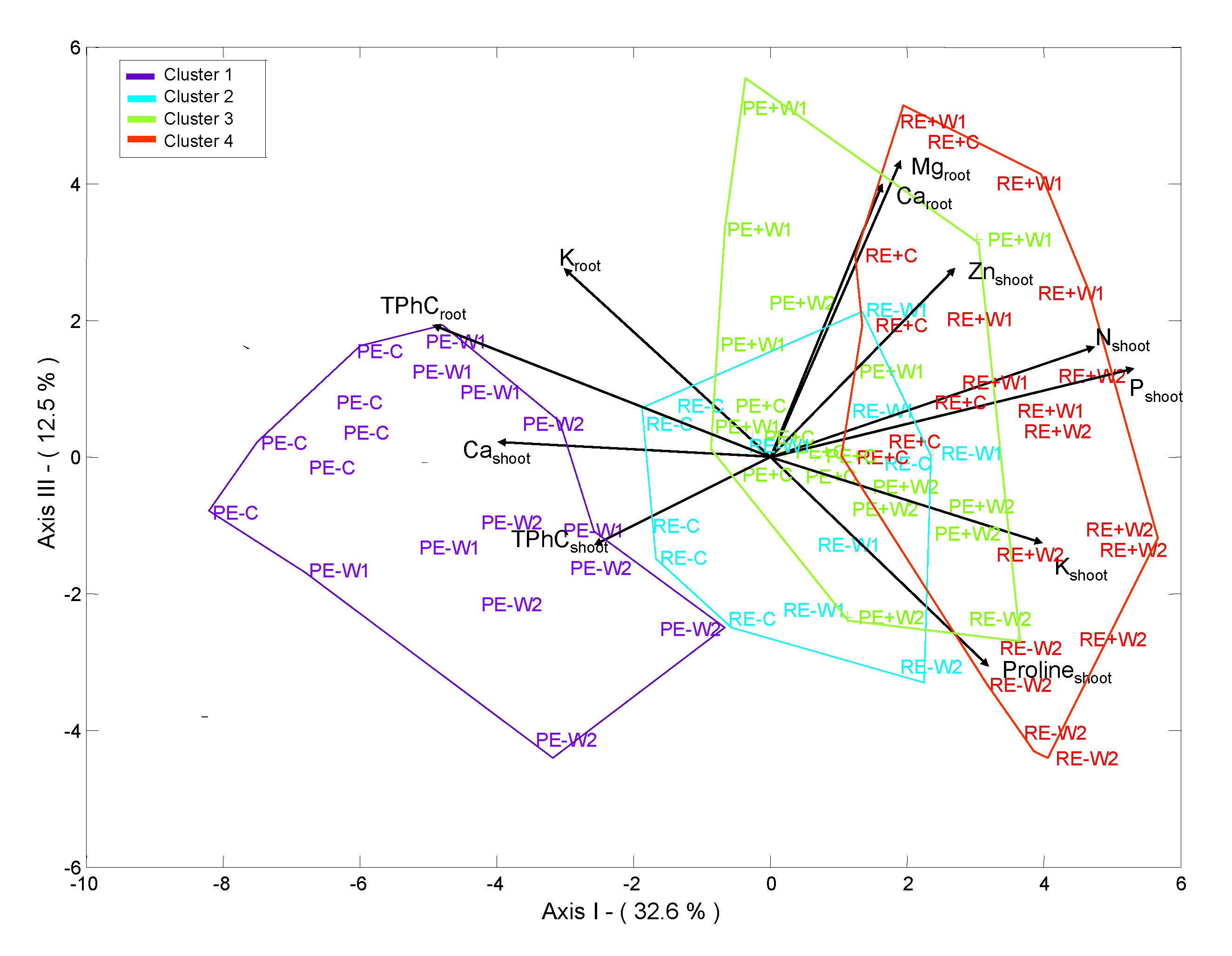

Supplement: Figure S1 — H-J biplot representation of samples and variables on the factorial plane I-III. Each sample was labelled with a first letter for plant line (P= PEN; R= RAB), a second letter for endophyte status (E+= endophyte infected; E-= non-infected), and a third letter for water treatment (C= control; W1= moderate stress; W2= severe stress). Variable labels: DWshoot = shoot biomass; DMroot=root biomass; TPhCshoot= total phenolic compounds in shoots; TPhCroot= total phenolic compounds in roots; Prolineshoot= proline in shoots; Prolineroot= proline in roots; and nutrient concentration in shoot and roots: Nshoot, Nroot, Pshoot, Proot, Kshoot, Kroot, Cashoot, Caroot, Mgshoot, Mgroot, Znshoot, Znroot. (TIF) [file pone.0084539.s001.tif]
